# Ethical issues in research on substance‐dependent parents: The risk of implicit normative judgements by researchers

**DOI:** 10.1111/bioe.12514

**Published:** 2018-09-18

**Authors:** Anke Snoek, Dorothee Horstkötter

**Affiliations:** ^1^ School for Mental Health and Neuroscience (MHeNS), Department of Health Ethics and Society, Maastricht University The Netherlands

**Keywords:** addiction, epistemic injustice, normative judgements, parental substance dependency, research ethics

## Abstract

When doing research among vulnerable populations, researchers are obliged to protect their subjects from harm. We will argue that traditional ethical guidelines are not sufficient to do this, since they mainly focus on direct harms that can occur: for example, issues around informed consent, fair recruitment and risk/harm analysis. However, research also entails indirect harms that remain largely unnoticed by research ethical committees and the research community. Indirect harms do not occur during data collection, but in the analysis of the data, and how the data is presented to the scientific and wider societal community. Highly stigmatized research subjects, like substance‐dependent parents, are especially at risk of encountering indirect harm, because the prejudice against them is so persistent. In this paper we discuss two forms of indirect harm. First, researchers have to be aware how their results will be preceived by society. Even when subjects are presented in an objective way, further, out of porportion stigmatization can occur. Researchers sometimes try to counteract this by whitewashing their results, at the risk of downplaying their respondents’ problems. The second risk researchers face is that their own normative judgements influence how they question such parents, report results and interpret statements. Researchers’ own normative judgements may influence the way they present their subjects. This article reviews a broad range of research that exhibits such indirect harms, discussing how and why indirect harms occur and formulating corresponding recommendations on how to prevent them.

## 1 TYPES OF HARM

Research participants from stigmatized vulnerable populations face three types of harm: traditional ethical issues, ethics in action and indirect harms. When doing research among vulnerable, highly stigmatized populations (like substance‐dependent parents), specific protective measures are required in order to provide adequate protection and reduce the risk of any further harm.^1^

Internationally approved standards and guidelines like the Declaration of Helsinki^2^ or the Guideline on Good Clinical Practice^3^ regulate today’s research with human participants. National laws give these guidelines a judicial basis. Ethical review boards in turn ensure that these ethical standards are upheld, ascertaining that contemporary research has scientific and/or social relevance and is set up according to sound methodological standards ensuring scientific validity. Ethical review boards also check that participants face an adequate risk–benefit balance, provide free and informed consent, have their privacy protected and get recruited fairly. Under these conditions participants are considered sufficiently protected against undue harm and researchers may expect ethics approval to conduct their projects unimpeded.

Despite its broad implementation, this system of ethics approval has been criticized for a number of reasons. A major criticism is that it would be unfit to evaluate certain kinds of research, most prominently qualitative research projects covering interviews and observational studies.^4^ Many ethical challenges cannot be anticipated in advance, but become apparent only concomitantly with research, or even retrospectively.^5^ This requires an ‘ethics in action’,^6^ in which researchers actively analyse and are ready to respond to possible ethical hazards rather than relying solely on approvals received beforehand. Holland and colleagues, for example, noticed that the child of a substance‐dependent parent got very upset during an interview. Upon direct engagement they found that the child had mistaken the scientist for a social worker coming to take him away.^7^ Such unintended and unforeseen harm to participants and family members is typical of qualitative research; it requires continuous attention and an immediate rectifying reaction independent of the research protocol and ethics approval.

Apart from these two direct harms (traditional anticipated research ethical issues and issues that occur during data collection), we will argue that indirect harms also occur, and that these are often overlooked.

First described by Munthe and colleagues,^8^ two types of indirect harm occur that current ethical standards are not sufficient to cover. The first type consists of harm from the implications of the study’s findings. Although research ethics is mostly concerned with preventing harm done during the phase of data collection, it is interesting to question whether ethical concerns should be expanded to unintended burdens that may occur even after the research is finished, for example due to policy changes that follow from the results of the study.

This type of harm most likely occurs in cases where research participants pose a risk to a third party, for example substance‐dependent parents might pose a risk to their children. Researchers can then face a role dilemma. On the one hand they have the obligation to protect their participants from undue harm. But on the other hand they also have to serve the public good and ensure that their research has social relevance.^9^ An example of this is if the findings contribute to new policies that are detrimental or disadvantageous to the participants. For example, research might discover that substance‐abusing parents are doing worse than currently assumed, leading to a new policy of stricter child custody measures. While this might increase child well‐being, parents might spiral down further due to the trauma of losing their children. Although children’s well‐being may overrule participants’ protection, this can cause an unintended burden to research participants, and they will be less likely to benefit from the knowledge, practices or interventions that result from the research.^10^

The main focus of this paper is the second type of harm, in which participants are indirectly harmed by the way they are represented to the research community and the wider society.^11^ This type of indirect harm is especially salient amongst stigmatized groups, because the prejudice against them is so persistent. Even when subjects are presented in an objective way,^12^ further, out‐of‐porportion stigmatization can occur. Barnard outlines how difficult doing justice to the struggles of substance‐dependent parents can be without further fuelling public stigma.^13^ She describes research as a balancing act between prejudice and whitewashing.^14^

Barnard describes two such balancing acts. The first is to determine how the researcher’s objective account will be received by the public. If they know their objective account will still fuel misguided stigma, should researchers whitewash their results? Or will this only downplay the respondents’ problems? The second balancing act is whether their account is objective or reflects researchers’ prejudices. Researchers might be ‘uncomfortable with the notion that they could implicitly or explicitly stand in judgement of [addicted] people’,^15^ particularly because this group is already socially excluded. However, doing research in such a value‐laden field involves these risks.^16^ The very fact that researchers are part of a community that holds serious prejudices against substance‐abusing parents can directly influence how they question such parents, report results and interpret statements.

An example of research bias against substance‐using parents, can be found in the so‐called ‘crack‐baby’ studies in the 1990s. Grave concerns about the effects maternal crack use had on fetuses resulted in studies that indeed revealed deficits in babies prenatally exposed to crack cocaine. However, when studies compared ‘crack babies’ to other babies from low socioeconomic backgrounds with a low birthweight, it turned out that ‘crack babies’ did not perform worse. Broader life circumstances including poverty and racial discrimination, and *not* drug usage, had been the main source of damages.^17^ Carl Hart recently debunked calls for stronger monitoring of marijuana usage in pregnant women as being based on bias rather than evidence. First, these calls are based on an overinterpretation of animal data. Doses used in animal studies are much higher than human medical or recreational doses. Second, cognitive deficits of children prenatally exposed to marijuana are overstated, and children are inappropriately labelled as impaired.^18^ He warns that ‘marijuana babies’ mirror the ‘crack baby’ debacle. Many feminist studies have shown that substance‐using mothers have been particularly demonized in contrast to fathers because they do not fit our image of ideal mothers.^19^ Babcock^20^ outlines how addiction research is mainly focused on maternal substance use, while studies indicating the influence of paternal substance use before conception on fetal health are ignored.^21^ Researchers are part of a societal system of unfair values that they risk reinforcing. Current research on implicit bias shows how hard, if not impossible, it is to overcome widely shared assumptions.^22^

Above we gave examples on how bias can influence research design, such as which factors are considered in the first place, and what questions get asked. In the following we will show how bias can lead to other, more subtle ways of misrepresenting research subjects. We analysed several research papers, and came across the following phenomena that repeatedly occurred and which risk inflicting indirect harm on research participants: (a) linking substance abuse to bad parenting automatically, rather than by argument or proper definition; (b) doubting the trustworthiness of the narratives of research participants; and (c) interpreting ambiguous findings in one‐sided ways that are excessively negative or positive, neither taking participants seriously. In the following we will analyse concrete examples of these problems.

## 2 IMPLICIT NORMATIVE JUDGEMENTS IN RESEARCH REPORTS

The idea for this article emerged when we were studying the literature on substance‐dependent parents for another project. We selected a wide range of articles on substance‐using parents for our literature exploration. When reading these articles, we came across many implicit judgements made by researchers in otherwise thoughtful articles. Such normative judgements do not directly follow from the data presented but rather reflect unconscious or pre‐reflective assumptions of the researchers themselves. We decided to collect these normative judgements in a separate file, and found at the end of our literature exploration that we identified many more text passages containing implicit normative judgements of researchers than we expected beforehand.

We want to emphasize that our analysis of the studies might give a distorted view of the papers. In general the research is done thoroughly and the language is non‐judgemental and non‐stigmatizing, except in the few places that we highlight. We believe that the possible harm done is unintended and unknown. This shows how easily normative judgements slip in when doing research among highly stigmatized groups. It is, therefore, important to raise awareness of these indirect harms done to vulnerable research participants in order to improve the situation of participants and support the research community. We finalize our analysis with several recommendations on how to recognize and avoid such indirect long‐term research harms and burdens.

Below are examples of these normative judgements, with indirect harms outlined as well as suggestions as to which implicit biases were in effect.

## 3 MAKING EXPLICIT THE LINK BETWEEN DEFICIENT PARENTING AND SUBSTANCE DEPENDENCY

The relationship between deficient parenting and substance dependency is complex. Although it is estimated that 50–80% of child welfare cases involve parental substance abuse,^23^ this is mediated by several other social and personal factors like poverty, mental illness and domestic violence.^24^ However, most articles implicitly assume a relationship between deficient parenting and substance abuse.^25^ Valentine and Treloar^26^ argue that ‘it is only rarely that the meaning of “alcohol or other drug issues” is examined in the context of parenthood’. Taylor and Kroll warn that researchers risk ‘making unfounded connections and assumptions between chronic substance misuse and problems in parenting’,^27^ reducing the complexity into a causal connection.

The following quote about whether the social network of substance‐dependent parents can support them in their parenting tasks shows this implicit connection between substance use and bad parenting:

There are now also concerns about whether grandparents will be able to care for grandchildren as some of these grandparents will themselves be substance users or have been substance users in the past.^28^

Russell, however, does not explain why grandparents who have been substance users in the past would be unable to take care of their grandchildren now. An underlying assumption may be that addiction is untreatable—once an addict always an addict. Alternatively they may assume that addiction is so devastating that even addicts who become clean will never be able to fulfill certain tasks and responsibilities. A clear understanding of the meaning of ‘substance usage’ and its long‐term consequences would help to clarify this point and explain rather than assume the unfitness of previous substance users as caregivers.

In their study of the practices of parents with mental illness and addiction, van der Ende and colleagues state: ‘In general, parenting varies from so‐called “good enough parenting” to “problematic parenting”.’^29^ This raises the question whether they truly did not find *any* examples of good parenting. Here, it is presented as a matter of fact that mental illnesses, including addiction, are incompatible with good parenting. It is problematic that the authors do not specify what they mean by ‘good enough parenting’ or ‘problematic parenting’. A lack of definition makes it hard to link parenting with any problems.^30^

One of the reasons that the assumed relationship between deficient parenting and substance dependency would be unfounded is the very fact that there seems to be very little consensus about what should be considered as irresponsible or problematic substance use^31^ and whether to call it abuse, dependency, addiction, a disease, and so on. There is ‘a bewildering array of ways of talking about excessive use of substances’,^32^ but most often clear definitions are absent altogether. Unexamined and unclear definitions^33^ make it hard, if not impossible, to draw any clear relationships between usage and parenting or child‐rearing abilities. Forrester and Harwin,^34^ provide an exception: they extensively discuss definitions (we will discuss this later on in Section 5).

Poor or absent definitions lead to presentation of research results that fail to distinguish between the severity of the effects of different substances on parenting. Subsuming all different substances under one heading of ‘addiction’ or ‘dependency’ disregards the different effects on parental capacities. The following quote shows this vividly:

Both the ethics committee and the research team took researcher safety very seriously in this project, developing clear lone worker protocols. Nonetheless, despite these concerns, few physically risky situations were encountered, with the researcher only deciding not to enter a home on one occasion due to concerns about potential risks posed by the strong smell of cannabis being smoked and a young man jumping out of the window and sprinting away from the home.^35^

We do not want to criticize the researcher’s feeling unsafe. If a researcher feels unsafe, for whatever reason, they should always be free to annul the interview. We do wonder though why the authors of the paper find it necessary to state that ‘research safety’ was taken ‘very seriously’. Hence, they present the parents they interview as potentially dangerous; even though the parents had volunteered to participate in the research project, thereby expressing some kind of trust in the researcher. Also the example they give of compromised safety is quite ambivalent. If they would have only described the person jumping out of the window as making the researcher feel physically unsafe, their example would be less ambivalent. We wonder, however, why the researcher felt unsafe because of the smell of cannabis. Some substances, like alcohol, amphetamines and cocaine, can increase violent tendencies in the user (although this relationship is not simply causal either)^36^ and researcher safety might be threatened. However, it is generally known that cannabis use is not linked to violence, rather the opposite is true.^37^ Hence, presenting cannabis use as a physical danger seems to misrepresent substance users. Also, running away could be a sign of the researcher being feared or avoided rather than that the fleeing person was to be feared. Apparently in this study, substance usage in general was considered a threat to the researchers, but the researchers do not specify why. If substance use is—in this case mistakenly—regarded as threatening in itself, will the researcher be able objectively to consider the effect of substance use on their respondents’ parenting skills?

Since the relationship between substance abuse and parenting is rather complex, and given the public consensus that substance abuse is incompatible with parenting, researchers risk making unfounded assumptions. This is exacerbated by the bewildering array of ways substance use is talked about, with no consensus on what irresponsible or responsible substance usage would entail.

## 4 DOUBTING THE TRUSTWORTHINESS OF THE RESEARCH SUBJECTS

A ‘scoring results’ workshop in the Netherlands in 2009 invited a woman who had overcome her substance dependency to share her experiences of horrible abuse within a treatment facility. At the end, the chairman responded with ‘*if it is true *what she is saying, we have much to learn from her story’ (our emphasis). This chairman very casually doubted the trustworthiness of the speaker.

Similar attitudes are rife in the ‘limitation’ sections of various qualitative research reports. We will argue that these ‘limitation’ sections do not harmlessly reflect a general doubt researchers have against self‐report, but that expressing doubt of trustworthiness so prominently—without providing evidence—can lead to further stigma, or even to what Miranda Fricker famously termed ‘epistemic injustice’. This form of epistemic injustice is called testimonial injustice, and occurs when ‘a speaker receives an unfair deficit of credibility from a hearer owing to prejudice on the hearer's part’.^38^ The result is that the subject, although possessing first‐person knowledge on the topic, is not taken sufficiently seriously in the process of knowledge formation.

Reports emphasize the importance of taking into account the voices of substance‐dependent parents, currently missing in the debate,^39^ but simultaneously they structurally doubt the trustworthiness of their respondents:

Important to note before turning to the findings is that this study focuses on women’s stories about their mothering practices and not on their actual practices.^40^

The research therefore benefited from an important, privileged and (we hope) honest insight into the day‐to‐day lives of parents who use drugs.^41^

[*Limitations of the study*] Fourth, steps to analyze trustworthiness with the scheme of Shenton (2004) were not executed.^42^

No justification is given by Baker and Carson for their assumption about a gap between the stories and the practices, nor by Elliott and Watson for only *hoping* for honesty rather than simply taking it for granted. Van der Ende and colleagues also undermine their findings by stating that they did not perform a trustworthiness check on their data. Authors thus contribute to reducing the impact and relevance of the voices of those directly concerned.

We acknowledge obvious limits to self‐reporting and discrepancies between actual events and people’s recollections do occur. However, researchers should explicitly outline their reasons for believing participants have reported inaccurately, misrepresented or overstated their parental capacities or their children’s situation.

Mentioning the credibility of vulnerable research populations like substance‐dependent parents is problematic if this is not, or to much lower degrees, expressed about self‐reports of, for example, professionals working with such parents. The chairman mentioned earlier was pointed towards this discrepancy by another participant, who objected that none of the other speakers (who had all been professionals) were similarly doubted. Moreover, published literature does not find similar ‘limitations of the study’ regarding professionals interviewed about their work and their views on substance‐abusing parents. Professionals’ stories are taken at face value.

Miranda Fricker’s illuminating work argues that researcher bias is not as harmless as it seems,^43^ but a form of *testimonial injustice*. This dysfunction is both epistemic and ethical. Epistemically, the interviewer overlooks the speaker’s essential information and knowledge; and ethically, the interviewee is wronged in his or her capacity as a knower, and excluded from the knowledge‐forming process. Several scholars outline how this form of injustice plays a role in modern healthcare practices^44^ and mental health services.^45^

Although many researchers aim to provide marginalized people with a voice, they simultaneously very subtly doubt the trustworthiness of their respondents. This leads readers to form or sustain an image of substance‐abusing parents as untrustworthy, even though these parents’ trustworthiness was not the topic of the study, and no evidence is provided for their suspected untrustworthiness. Doubting the trustworthiness of respondents also wrongs them as knowledgeable research subjects.^46^

## 5 INTERPRETING AMBIVALENCE IN THE STORIES OF RESEARCH SUBJECTS

Narrative accounts can contain contradictory elements. How researchers interpret these ambivalences can also reveal researchers’ implicit normative assumptions which bias interpretation of the subjects, confirming assumptions rather than being open minded towards participants’ reports. We will give three examples of this.

Wolf and Chavez attempt to justify questioning respondents’ reliability by arguing that stigma may have hindered their respondents from speaking freely. They similarly justify when they think their accounts have been truthful:

[A]s parental substance use is a stigmatized behavior (…), parents may not have been truthful about their alcohol‐related behaviors or beliefs, producing social desirability bias. This could partially explain why many of our themes suggest that drinking around children is perceived as negative by parents. While we are unable to gauge the extent of social desirability bias in our findings, several parents did report drinking very large quantities of alcohol (approximately 9 beers a day), suggesting that they were answering questions openly.^47^

While this demonstrates how researchers can avoid problems discussed in the previous section (they explicitly state why they think the parent’s accounts were reliable or not), another implicit normative judgement slid into their account. Wolf and Chavez did not understand why parents stated that they prefer not to drink in the presence of their children. Parents’ statements did not match the researchers’ expectations of them. The authors assume that parents said this out of social desirability, especially because they did report drinking when their children were around. However, people can fail to act on what they say they value for two reasons. The first explanation is a moral one: they might not really value what they say they value. Actions speak louder than words. We determine what people really value by what they do, not at what they say. Although parents say they value their children more than drinking, the fact that they drink around their children shows otherwise. The second explanation comes from neuroscience. Robinson and Berridge show that wanting and valuing are mediated by different neural pathways. In addiction these pathways often come apart, resulting in a strong craving in absence of valuing.^48^ Parents may value not drinking around their children, but due to craving still do so. The researchers seem to opt for the first, moral, explanation: subjects explicitly and repeatedly state that they do not value drinking around their children only because of social desirability bias rather than because they have separate wants and values.

In the second example the researcher considers any ambivalence as a sign of untrustworthiness or inconsistency:

Even talking retrospectively, Emily’s account *was contradictory*. At one point Emily claimed the drugs were the priority and controlled her life, later Emily claimed she had the control over the drugs by having a routine in her use. For example, Emily tried implementing a boundary to her crack use, she set a time before which she would not start smoking crack, almost self‐reassurance that she had some control over the crack.^49^

By presenting this as a sign of contradiction, Melhuish undermines the respondent’s trustworthiness or sincerity. However, such discrepancies could alternatively be explained by the fact that addiction has different stages in which control indeed varies.^50^ The woman cited could be describing different phases in which she had highly varying control over her addiction. Alternatively, control may alter throughout the day. In either case, the report would contain no contradiction, but a fair description of different situations at different times.

Researchers’ struggle with perceived or real ambivalence is a sign of epistemic injustice. Fricker calls this *hermeneutic injustice*, which occurs when a researcher fails to understand a certain social phenomenon due to a lacuna in the collective understanding of the phenomenon. The result is that interpretations are structurally prejudiced, and respondents are not heard.^51^ Instead of asking their respondents to explain the ambivalence, the researchers assume that they are untrustworthy.

Implicit normative judgements can lead to depreciating interpretations of ambivalent research findings, yet the opposite can also occur. Researchers can be so dedicated to destigmatizing and empowering substance‐dependent parents that results are whitewashed, putting participants in the most benevolent light,^52^ while neglecting the more problematic aspects of their respondents’ stories. Baker and Carson, similar to Melhuish, concluded that mothers presented a contradictory picture of the quality of their mothering practices, revealing both serious neglect and endangerment of their children, and periods of responsible and loving mothering practices. However, in an attempt to counteract the current demonization of addicted parents, they emphasize that these women are caring and committed mothers:

many studies fail to recognize how these women really care about their children and try hard to balance their use and that care. As a result … the depiction of their mothering practices may be inaccurate and more negative than appropriate or valid.^53^

However, Baker and Carson’s account may be too optimistic. The mothers they have interviewed appear to have the best intentions, deploy harm‐reduction strategies, and perceive ‘themselves as good moms, in some aspects or another, even when they were using’.^54^ The authors conclude that substance‐dependent mothers can be good mothers, at least some of the time. But what is missing is a fair emphasis on both good and insufficient mothering practices. Their respondents also describe episodes of drunk driving and causing accidents while their children were in the car and their children viewing and experiencing violence due to their substance‐using lifestyle. Whether the episodes of good parenting outweigh the bad episodes remains unresolved. Baker and Carson suggest that they do, and that these mothers are judged harshly because the dominant model of motherhood is intensive parenting. This optimistic representation does not sufficiently acknowledge the parents’ struggles, and this might hinder parents in getting needed support.

Often, researchers cannot unambiguously reveal which interpretation of specific respondents’ reporting on the situation is most apt. However, in order to prevent indirect harm to research participants both pejorative and euphemizing interpretations must be avoided. Research reports should explicitly remain open‐minded and offer various relevant scenarios to avoid biased assumptions. We acknowledge that this is especially hard in such morally loaded research contexts.

## 6 WAYS OUT AND RECOMMENDATIONS

The problems described above occur because of implicit normative judgements held by researchers. Thus researchers are neither conscious of their own judgements, nor do they intentionally report, or omit, research findings in a misleading way. Given that all people hold a variety of implicit biases, it would be inadequate to blame researchers for having what is a psychological fact of life. Consequently, working in a morally loaded territory, such as substance‐dependent parenting, is particularly challenging in this respect. However, there are things researchers can do to try to counteract their implicit judgements.

In the following, we would like to recommend ways to become aware of bias and then avoid pejorative or whitewashing assumptions. This will both reduce indirect harm inflicted on parents who take part in research projects, and improve the quality of research reporting. These recommendations are not only relevant for researchers, but also for peer reviewers.

Regarding unfounded assumptions connecting insufficient parenting with substance abuse, we recommend both explicitly defining substance abuse, and explicating the connection between substance abuse and insufficient parenting. Forrester and Harwin outline how defining substance dependency is also important for examining our own implicit normative assumptions:

[T]he terminologies [about substance dependency] involve different sets of assumptions, beliefs and values about excessive substance use. They therefore provide an important starting point for considering our own values and assumptions. For instance, a professional who calls someone an ‘alcoholic’ is—whether they are aware of it or not—making different assumptions from one who says the same individual has an ‘alcohol problem’. Informed practice therefore starts with a consideration of the words we use and the models they relate to.^55^

Careful language examination reduces the risk of making unfounded assumptions. Researchers struggling with this could read Forrester and Harwin’s study which offers many suggestions on how to define substance abuse.^56^

The second risk, implicitly undermining the trustworthiness of our respondents, could be reduced if researchers were more careful when stating that self‐report is untrustworthy. Reasons to doubt subjects’ testimony should be clearly outlined.

Regarding the third risk, several exemplar studies show how researchers can interpret the ambivalence within the stories of research subjects without relying on implicit or explicit normative judgements. Barnard argues that one should not look for an ‘objectified definitive “truth”’,^57^ but should study the processes involved, even when accounts are contradictory. Phases of use should get distinguished to this end. Rhodes and colleagues demonstrate the fruitfulness of this approach. They have parents outline the effectiveness of their harm‐reduction strategies, especially how these were adjusted as their children aged. These in‐depth interviews give detailed insight into how parents tried to limit the damage, and how this did not always work.^58^ This gave a more realistic view of the struggle of substance‐dependent parents in contrast to a more binary approach (parents have control or they don’t). Another method is for researchers simply to ask subjects to explain ambivalence. Asking subjects to validate the analysis once it is done is also beneficial.^59^

When encountering ambivalence in the respondents’ stories, researchers should be cautious about interpretation. Does ambivalence reflect their own expectations of what the subjects would say? Or can subjects explain what researchers perceive as ambivalence? Researchers should be aware of their own normative agenda^60^ and that different stages of use involve different levels of control.

## 7 CONCLUSION

When studying vulnerable, highly stigmatized groups, complex ethical issues arise beyond those that aim at avoiding direct harm. Indirect harms occur: namely subjects can be harmed by how they are represented in research reporting. Even representing our subjects in an objective matter can still lead to unfair stigma. It is difficult to perform research in such morally laden territory. Although researchers aim at improving the care for these families, all too easily, normative assumptions seep into their research, resulting in further stigmatization of addicted parents, further aggravating the problems these parents face.

In this article, we highlight some of the implicit normative judgements made by researchers on parental substance abuse. We discuss: (a) the importance of defining substance abuse and making explicit the influence of substance abuse on parental capacities; (b) how to handle the trustworthiness of self‐report; and (c) how to deal with ambivalence in respondent’s stories. We offer some advice on how these normative judgements can be avoided. We think our findings are also relevant for studies among other vulnerable groups.

We also challenge current research ethical frameworks that mostly focus on the protection from undue harms and burdens during the phase of data collection and while study procedures are taking place. Our findings reveal that harm or burdens can also be inflicted on research participants in the long term, that is, long after data collection has been completed. Such indirect burdens can arise from the way in which research findings get presented and published by academic authors. In order to ensure that research is done in an ethical way, researchers involved in research with addicted parents, and probably also other vulnerable groups, should pay attention not only to the protection of participants while the study goes on, but also keep their interests in mind in the long run. This, however, requires that researchers extend their ethical attitudes beyond what current research ethical guidelines expect from ethical research and ethical researchers.

## CONFLICT OF INTEREST

The authors declare no conflict of interest.

**A** **nke** **S** **noek**, PhD, is a post‐doctoral researcher at the School for Mental Health and Neuroscience (MHeNS), Department of Health, Ethics and Society, at Maastricht University, The Netherlands. Her research interests cover the agency of marginalized groups. Her work covers the intersection of neuroscience, lived experiences, and narrative self‐conception.

**D** **orothee** **H** **orstkötter**, PhD, is assistant professor at the School for Mental Health and Neuroscience (MHeNS), researcher at the Department of Health, Ethics and Society, at Maastricht University, The Netherlands. Her research interests cover ethical and conceptual questions at the interface of genomics, neurobiology, social psychology and (disordered) human behaviour.

## Footnotes

1 Anderson, E. E., & DuBois, J. M. (2007). The need for evidence‐based research ethics: A review of the substance abuse literature. *Drug and Alcohol Dependence*, *86*(2–3), 95–105.

2 World Medical Association. (2013). *Declaration of Helsinki, Ethical principles for medical research involving human subjects*. Retrieved from http://www.wma.net/en/30publications/10policies/b3/

3 ICH. (1996). *Guideline for good clinical practice*. Retrieved from https://www.ich.org/fileadmin/Public_Web_Site/ICH_Products/Guidelines/Efficacy/E6/E6_R1_Guideline.pdf

4 Holland, S., Williams, A., & Forrester, D. (2014). Navigating ethical moments when researching substance misuse with parents and their children. *Qualitative Research*, *14*(4), 411–427.

5 Hugman, R. (2010). Social work research and ethics. In I. Shaw, K. Briar‐Lawson, J. Orme, & R. Ruckdeschel (Eds.), *The SAGE handbook of social work research* (pp. 149–163). London, UK: Sage; Gabb, J. (2010). Home truths: Ethical issues in family research home truths. *Qualitative Research*, *10*(4), 461–478; Iphofen, R. (2011). Ethical decision making in qualitative research. *Qualitative Research*, *11*(4), 443–446; Holland et al., *op. cit.* note 4.

6 Holland et al., *op. cit.* note 4.

7 Ibid.

8 Munthe, C., Radovic, S., & Anckarsäter, H. (2010). Ethical issues in forensic psychiatric research on mentally disordered offenders. *Bioethics*, *24*(1), 35–44.

9 Ibid.; Robertson, M. D., & Walter, G. (2008). Many faces of the dual‐role dilemma in psychiatric ethics. *Australian and New Zealand Journal of Psychiatry*, *42*, 228–235.

10 It should be noted that thereby research of this type might as a matter of principle challenge the requirements of the internationally acknowledged Declaration of Helsinki, which explicitly states that vulnerable groups ‘should stand to benefit from the knowledge, practices or interventions that result from the research’. Apparently, here the Declaration of Helsinki mainly wants to avoid the inclusion of vulnerable groups in so‐called non‐therapeutic research, that is, research from which participants can never profit individually—as opposed to so‐called therapeutic research. Our concerns indeed go beyond this context. However, one should keep in mind that the Declaration of Helsinki aims only at the protection of participants during the phase of data collection and ignores any harms or burdens that might result in the long term. WMA. (2013). Declaration of Helsinki. *Bulletin of the World Health Organization*, *79*(4), 373–374.

11 Munthe et al., *op. cit.* note 8.

12 Appelbaum, P. S. (1997). A theory of ethics for forensic psychiatry. *Journal of the American Academy of Psychiatry and the Law*, *25*(3), 233–247.

13 One could argue that the risks that the children of substance‐dependent parents face justify treating the parents with distrust and even stigma. However, studies have shown that the stigma that the parents face harms the children as well. Garcia, S. A. (1993). Maternal drug abuse: Laws and ethics as agents of just balances and therapeutic interventions. *Substance Use and Misuse*, *28*(13), 1311–1339; Burk, J., & Sher, K. (1988). The 'forgotten children' revisited: Neglected areas of COA research. *Clinical Psychology Review*, *8*, 285‐302; Toufexis, A. (1991, May 13). Innocent victims. *Time*, pp. 56–60.

14 Barnard, M. (2005). Discomforting research: Colliding moralities and looking for ‘truth’ in a study of parental drug problems. *Sociology of Health and Illness*, *27*(1), 1–19.

15 Ibid.,p. 2.

16 Ibid.; Baker, P. L., & Carson, A. (1999). ‘I take care of my kids’: Mothering practices of substance‐abusing women. *Gender & Society*, *13*(3), 347–363; Taylor, A., & Kroll, B. (2004). Working with parental substance misuse: Dilemmas for practice. *British Journal of Social Work*, *34*(8), 1115–1132.

17 Frank, D. A., Augustyn, M., Knight, W. G., Pell, T., & Zuckerman, B. (2001). Growth, development and behavior in early childhood following prenatal cocaine exposure: A systematic review. *Journal of the American Medical Association*, *285*(12), 1613–1625; Glenn, J. E. (2014). Making crack babies: Race discourse and the biologization of behavior. In L. B. Green, J. Mckiernan‐González, & M. Summers (Eds.), *Precarious prescriptions:* *Contested histories of race and health in North America *(pp. 237–260). Minneapolis, MN: University of Minnesota Press; Hurt, H., Giannetta, J. M., Korczykowski, M., Hoang, A., Tang, K. Z., Betancourt, L., … Detre, J. A. (2008). Functional magnetic resonance imaging and working memory in adolescents with gestational cocaine exposure. *Journal of Pediatrics*, *152*(3), 371–377.

18 Stadterman, J. M., & Hart, C. L. (2015). Screening women for marijuana use does more harm than good. *American Journal of Obstetrics & Gynaecology*, *213*(4), 598–599; Torres, C. A., & Hart, C. L. (2017). Letter to the Editors. Marijuana and pregnancy: Objective education is good, but biased education is not. *American Journal of Obstetrics & Gynaecology*, *217*(2).

19 Olsen, A. (2015). Punishing parents: Child removal in the context of drug use. *Drug and Alcohol Review*, *34*(1), 27–30; Valentine, K., & Treloar, C. (2013). Response to Chandler et al. Substance, structure and stigma: Parents in the UK accounting for opioid substitution therapy during the antenatal and postnatal periods. *International Journal of Drug Policy*, *24*(6), e87–e88.

20 Babcock, M. (2008). Substance‐using mothers: Bias in culture and research. *Journal of Addictions Nursing*, *19*(2), 87–91.

21 Research is biased towards clinical populations with a low socioeconomic background which have the worse outcomes, and inform our opinions on addiction as a chronic, relapsing disorder. However, the white middle class display more control over use: McCoy, K., McGuire, J., Curtis, R., & Spunt, B. (2005). White chicks on dope: Heroin and identity dynamics in New York in the 1990s. *Journal of Drug Issues*, *32*(April), 817–842. Research on the general population reveals increased spontaneous recoveries: Heyman, G. M. (2009). *Addiction: A disorder of choice*. Cambridge, MA: Harvard University Press.

22 Brownstein, M. (2017). Implicit bias. *Standford Encyclopedia of Philosophy.* Retrieved from https://plato.stanford.edu/archives/spr2017/entries/implicit‐bias/

23 Niccols, A., Milligan, K., Smith, A., Sword, W., Thabane, L., & Henderson, J. (2012). Integrated programs for mothers with substance abuse issues and their children: A systematic review of studies reporting on child outcomes. *Child Abuse and Neglect*, *36*(4), 308–322.

24 Klee, H. (1998). Drug‐using parents: Analysing the stereotypes. *International Journal of Drug Policy*, *9*(6), 437–448; Olsen, *op. cit. *note 19; Valentine & Treloar, *op. cit. *note 19.

25 Victor and colleagues (2018) have outlined that many child welfare agencies do not consider parental substance misuse in and of itself to constitute child maltreatment, but require harm or threat of harm to be present in order for parental substance misuse to meet the official threshold for abuse or neglect. However, when analysing 501,060 substantiation decisions, of cases in which the reported maltreatment was not linked to parental substance misuse, they found that identified parental substance misuse nearly tripled the probability of substantiation. They suspect a bias from child welfare workers against these families. It seems that the bias against these families is ingrained in many levels of society. Victor, B. G., Grogan‐Kaylor, A., Ryan, J. P., Perron, B. E., & Gilbert, T. T. (2018). Domestic violence, parental substance misuse and the decision to substantiate child maltreatment. *Child Abuse and Neglect*, *79*, 31–41.

26 Valentine & Treloar, *op. cit. *note 19.

27 Taylor & Kroll, *op. cit. *note 16, p. 1116.

28 Russell, P. (2006). *Have we got our priorities right? Children living with parental substance use* (p. 29). Stirling, UK: Aberlour Childcare Trust.

29 van der Ende, P. C., van Busschbach, J. T., Nicholson, J., Korevaar, E. L., & van Weeghel, J. (2016). Strategies for parenting by mothers and fathers with a mental illness. *Journal of Psychiatric and Mental Health Nursing*, *23*(2), 87.

30 Thanks to an anonymous reviewer for pointing out that ‘good enough’ parenting could refer to Winnicott’s use of the term, which is in fact considered to be good parenting, and is contrasted with going beyond ‘good enough’—which can amount to helicopter parenting, parenting striving for unattainable ideals etc. However, the authors do not refer to Winnicott anywhere in their paper. Winnicott, D. W. (1965). *The maturational processes and the facilitating environment: Studies in the theory of emotional development.* New York, NY: International Universities Press.

31 Olsen, *op. cit. *note 19.

32 Forrester, D., & Harwin, J. (2002). What is ‘substance misuse’. In *Parents who misuse drugs and alcohol. Effective interventions in social work and child protection* (pp. 9–29). Chichester, UK: Wiley‐Blackwell, p. 9.

33 Valentine & Treloar, *op. cit. *note 19.

34 Forrester & Harwin, *op. cit. *note 32.

35 Holland et al., *op. cit.* note 4, p. 419.

36 Boles, S. M., & Miotto, K. (2003). Substance abuse and violence: A review of the literature. *Aggression and Violent Behavior*, *8*(2), 155–174; Chermack, S. T., & Giancola, P. R. (1997). The relation between alcohol and aggression: An integrated biopsychosocial conceptualization. *Clinical Psychology Review*, *17*(6), 621–649.

37 Smith, P. H., Homish, G. G., Collins, R. L., Giovino, G. A., White, H. R., & Leonard, K. E. (2014). Couples’ marijuana use is inversely related to their intimate partner violence over the first 9 years of marriage. *Psychology of Addictive Behaviors*, *28*(3), 734–742.

38 Fricker, M. F. (2007). *Epistemic injustice. Power & the ethics of knowing* (p. 9). Oxford, UK: Oxford University Press.

39 E.g. Baker & Carson, *op. cit.* note 16; Melhuish, J. (2011). Crack cocaine use and parenting: An analysis of three parents’ accounts of the impact their crack cocaine use had on family life. *Practice: Social Work in Action*, *23*(4), 201–213.

40 Baker & Carson, *op. cit.* note 16, p. 350.

41 Elliott, E., & Watson, A. (2002). Responsible carers, problem drug takers or both? In F. Harbin & M. Murphy (Eds.), *Substance misuse and child care. How to understand, assist and intervene when drugs affect parenting* (pp. 27–39). Dorset: Russell House Publishing, p. 28.

42 van der Ende et al., *op. cit. *note 29, p. 94.

43 Fricker, *op. cit.* note 38.

44 Buchman, D. Z., Ho, A., & Illes, J. (2016). You present like a drug addict: Patient and clinician perspectives on trust and trustworthiness in chronic pain management. *Pain Medicine*, *0*(January), 1–13; Kidd, I. J., & Carel, H. (2017). Epistemic injustice and illness. *Journal of Applied Philosophy*, *34*(2), 172–190.

45 Lakeman, R. (2010). Epistemic injustice and the mental health service user. *International Journal of Mental Health Nursing*, *19*(3), 151–153.

46 An example of epistemic injustice can also be found in the following quote. When research subjects explicitly try to distinguish themselves from the prevelant stigmatized image that they encounter, the researchers label them ‘in denial’, rather then exploring their different view, or providing evidence of why they think the subjects resemble the stigmatized image rather than their own description of themselves: ‘It was clear that John and Matthew rejected the negative stereotypes of drug users. Matthew appears to not want to be labelled in this negative way. John similarly draws a distinction between himself and other users: “I’ve never been desperate, I’ve never been a desperate drug taker.” Matthew and John were conscious of how they were viewed by society. The participants tried to draw distinctions between themselves and other drug users which could perhaps be attributed to a lack of acceptance and denial of the position they were in.’ Melhuish, *op. cit. *note 39, p. 206.

47 Wolf, J. P., & Chavez, R. (2015). ‘Just make sure you can get up and parent the next day’: Understanding the contexts, risks, and rewards of alcohol consumption for parents. *Families in Society*, *96*(3), 231.

48 Berridge, K. C., Robinson, T. E., & Aldridge, J. W. (2009). Dissecting components of reward: ‘Liking’, ‘wanting’, and learning. *Current Opinion in Pharmacology*, *9*(1), 65–73.

49 Melhuish, *op. cit.* note 39, p. 206 (author’s emphasis).

50 Barnard, *op. cit. *note 14.

51 Fricker, M. F. (2008). Forum on Miranda Fricker’s epistemic injustice: Power and the ethics of knowing précis. *Theoria*, *4548*(61), 69.

52 Barnard, *op. cit. *note 14.

53 Baker & Carson, *op. cit.* note 16, p. 356.

54 Ibid., p. 367.

55 Forrester & Harwin, *op. cit. *note 32, p. 9.

56 We acknowledge that we didn’t define substance abuse either in this study.

57 Barnard, *op. cit. *note 14, p. 1.

58 Rhodes, T., Bernays, S., & Houmoller, K. (2010). Parents who use drugs: Accounting for damage and its limitation. *Social Science & Medicine*, *71*(8), 1489–1497.

59 Wertz, F. J., Charmaz, K., McMullen, L. M., Josselson, R., Anderson, R., & McSpadden, E. (2011). *Five ways of doing qualitative analysis: Phenomenological psychology, grounded theory, discourse analysis, narrative research, and intuitive inquiry*. New York, NY: The Guilford Press.

60 In Shenton’s guide for enhancing trustworthiness in research, one of the steps is ‘Admission of researcher’s beliefs and assumptions’. Shenton, A. K. (2004). Strategies for ensuring trustworthiness in qualitative research projects. *Education for Information, 22*(2), 63–75.

